# Healthcare utilisation and cost expenditures for pneumonia in individuals with diabetes mellitus in the USA

**DOI:** 10.1017/S0950268819000979

**Published:** 2019-05-29

**Authors:** K. Liu, G. C. Lee

**Affiliations:** 1 The University of Texas at Austin, College of Pharmacy, Austin, TX, USA; 2 The University of Texas Health San Antonio, School of Medicine, San Antonio, TX, USA

**Keywords:** Diabetes, epidemiology, pneumonia

## Abstract

Pneumonia is one of the leading causes of hospitalisations among adults in the USA. Individuals with diabetes mellitus (DM) have been associated with increased risk for pneumonia and complications including death. The objectives of this study were to (1) compare the prevalence and healthcare utilisation patterns for pneumonia in individuals with and without DM, and (2) identify risk factors for pneumonia in those with DM. We performed a retrospective, cross-sectional analysis of the US adult population using Medical Expenditure Panel Surveys (MEPS) data from 2014. Overall, the data represented 24 million individuals with DM and 218 million without DM in the USA. The population-based rate for a pneumonia event was 34 per 1000 persons for individuals with DM and 19 per 1000 persons without DM. Compared to the non-DM group, individuals with DM were treated 1.8x, 2.6x and 1.4x more in the ED, hospital and outpatient, respectively. Furthermore, the average cost per pneumonia event was significantly higher among individuals with DM compared to non-DM in the inpatient setting ($11 931 *vs.* $7751; *P* < 0.001). Among individuals with DM, female sex, DM complications, smokers and administration of pneumococcal vaccines were significant factors associated with a pneumonia event.

## Introduction

Diabetes mellitus (DM) is one of the leading causes of morbidity and mortality globally. Approximately 10% of the US population has DM [1, 2]. The association of DM and the increased risk for infections has long been established in the clinical community [3, 4]. Among individuals with DM, pneumonia is the most common infection managed in the hospital and the third most common infection treated in the emergency department (ED) [2]. Several studies have described the association of DM and increased risk for pneumonia [5, 6]. Individuals with DM may have increased susceptibility to pneumonia based on various factors, including dysfunctional immunity related to the harmful effects of hyperglycaemia, risk of aspiration, impaired lung function and other co-existing comorbidities [3, 4, 7]. However, there are limited data on the relationship between DM and risk for pneumonia, particularly as it applies to different populations and health care settings [5, 8]. The objectives of this study were to (1) compare the prevalence and healthcare utilisation patterns for pneumonia in individuals with and without DM, and (2) identify risk factors for pneumonia in those with DM.

## Methods

### Data source

We analysed data from the Medical Expenditure Panel Survey (MEPS), a programme of the Agency for Healthcare Research and Quality (AHRQ). MEPS is a set of a comprehensive survey that provides national estimates of healthcare use, expenditures, payments and insurance coverage. It collects information from individual households, medical providers and employers in the USA. Data are collected in an overlapping panel design, with respondents undergoing five rounds of interviews in a two-calendar year period.

There are three main components to MEPS: The Household Component (HC), Medical Provider Component and Insurance Component. The MEPS HC includes data from household respondents with additional information supplemented from the Medical Provider Component. The HC provides person-level data on the demographics, health conditions, health status, healthcare use and payment for a representative sample of the non-institutionalised civilian population. Each event in the file represents a unique household-reported medical event, and details such as date of visit, type of care and condition codes are provided. The HC data organise medical events into sub-components of healthcare settings; three of the sub-components used in this work were ‘Hospital Inpatient Stays’, ‘Emergency Room Visits’ and ‘Outpatient Department Visits’. In addition, we accessed data from the Diabetes Care Survey (DCS), one of the five HC supplemental paper questionnaires provided to respondents with a self-reported diagnosis of DM.

### Study design and definitions

We performed a retrospective, cross-sectional analysis of pneumonia prevalence, risk factors and healthcare utilisation patterns of individuals with DM, without DM and with DM-complications in 2014. In the MEPS interviews, survey respondents' health conditions were coded using the Clinical Classification Software (CCS) codes. The three healthcare settings studied were hospital inpatient, ED and outpatient clinics. Demographic characteristics, comorbidities and variables for diabetes management were extracted using the MEPS HC. Adults aged 18 years and older were included in the study. Individuals with DM were identified as those with a CCS code 049 or 050. A pneumonia event was defined with a CCS code 122. Individuals with DM-complications were defined as DM with documented retinopathy or nephropathy. Expenditures in MEPS represent amounts actually paid for health care services, including those collected for hospital inpatient care, ambulatory care provided in offices or hospital outpatient facilities, and care provided in the ED reported in the HC of the survey. Total expenses for a pneumonia event are defined as the sum of direct payments by households, private insurance, Medicare, Medicaid and other sources to providers of the care.

### Data and statistical analyses

Population-based pneumonia prevalence rates were calculated as the annual number of pneumonia events divided by the corresponding US non-institutionalised population. Population denominators were derived from the MEPS HC. The MEPS estimate US populations based on sampled persons in the target population (civilian non-institutionalised) for the entire year. Prevalence estimates were calculated to compare total pneumonia events and healthcare utilisations among the different healthcare settings between the subgroups. Multivariable logistic regression models were used to identify independent factors associated with pneumonia among individuals with diabetes. This model contained age, sex, comorbidities (e.g. chronic heart disease, emphysema, chronic bronchitis, cancer, tobacco use), presence of diabetes complications, factors related to diabetes care (vaccines, medications) and duration of the disease. Generalised linear models were used to adjust for demographic and clinical characteristics to estimate adjusted mean expenditures for pneumonia. To account for MEPS' complex study design, all analyses were adjusted using weights, clustering and stratification. SPSS 24.0^®^ (IBM Crop, Armonk, NY, USA) was used for all statistical analyses. Statistical significance was calculated using a *P*-value of <0.001.

## Results

The data represented 24 million individuals with DM and 218 million without DM in the USA. A quarter of the individuals with DM had DM-complications. Table 1 compares the cohort characteristics. Overall, individuals with DM were older (66 *vs.* 52 years of age). Sex, race, educational level and annual income were distributed similarly between individuals with and without DM. There were a higher proportion of individuals with DM with comorbid health conditions compared to those without DM, including hypertension (78% *vs.* 29%) and hypercholesteraemia (74% *vs.* 26%). Among individuals with DM, the mean duration of DM diagnosis was 13.6 years. More than half of individuals with DM (62%) were on oral medication for DM management and a quarter (24%) were on insulin. Less than half of individuals with DM (49%) reported receiving an influenza vaccination during 2014 and 61% reported a history of receiving a pneumococcal vaccine.

**Table 1.** Baseline demographics

Characteristics	DM population USA	%	Non-DM population USA	%
Total population	24 314 201	–	217 547 699	–
18–64	13 333 418	55	182 435 945	84
65 or older	10 980 783	45	35 111 754	16
Gender				
Male	11 641 677	48	104 196 980	48
Female	12 672 524	52	113 350 720	52
Race				
White	18 299 227	75	172 859 876	80
Black	3 795 210	16	25 274 538	12
Hispanic	3 678 116	15	32 951 641	15
Asian	1 413 215	6	12 756 701	6
Native American/Alaskan	265 156	1	1 212 647	1
Hispanic	3 678 116	15	32 951 641	15
Non-Hispanic	20 636 084	85	8 465 448 691	85
Annual income				
Below poverty line 2014	6 897 611	28.4	55 525 101	25.5
Above poverty line	17 412 653	71.6	161 839 734	74.5
Tobacco status				
Current smoker	2 717 931	11.2	28 407 482	13.1
Insurance status				
Private	2 896 936	55.7	20 931 040	69.5
TriCare	260 187	5	1 037 242	3.4
Medicare	3 675 068	70.7	13 411 843	44.5
Medicaid	746 757	14.4	2 205 693	7.3
Uninsured	156 273	3	1 727 735	5.7
Health condition				
Hypertension	18 899 141	78	62 209 232	29
Hypercholesterolemia	17 890 977	74	57 359 796	26
DM-related characteristics				
DM-associated retinopathy	3 663 917	15.1	–	–
DM-associated nephropathy	2 225 573	9.2	–	–
Uses insulin	5 924 648	24.4	–	–
Use oral anti-DM medications	15 207 362	62.5	–	–

The overall population-based rate for a pneumonia event was 34 per 1000 persons for individuals with DM compared to 19 per 1000 persons for individuals without DM. Among individuals with DM, the population-based rate for pneumonia among those with DM-complications was twofold greater than those with DM without complications (60 per 1000 person *vs.* 28 per 1000 persons). Among individuals with DM, female sex (OR 2.1 (95% CI 2.08–2.10)), smokers (1.33, 1.30–1.36), presence of DM complications (1.15, 1.14–1.16) and history of pneumococcal vaccine (1.07, 1.07–1.08) were significant factors associated for a pneumonia event.

**Fig. 1.**
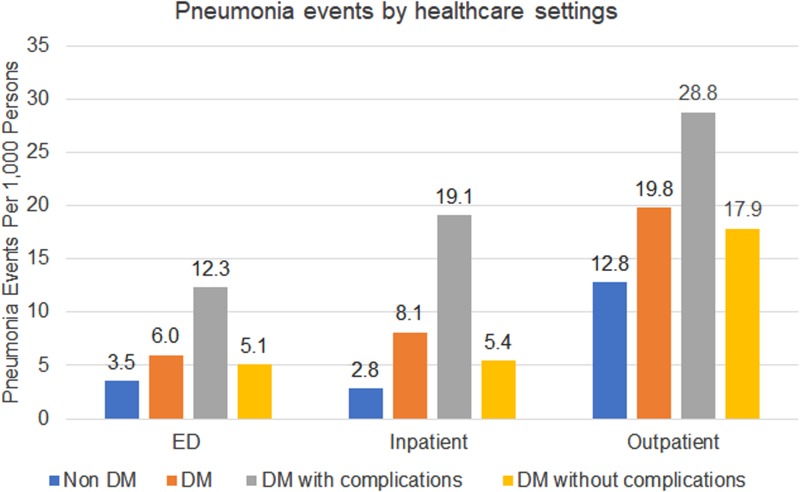
Pneumonia events by healthcare settings.

There were substantial differences in healthcare utilisation by setting among those with and without DM (Fig. 1). Compared to the non-DM group, individuals with DM were treated 1.8x, 2.6x and 1.4x more in the ED, hospital and outpatient, respectively. Among individuals with DM, those with DM-complications had higher healthcare utilisation across all healthcare settings: ED 1.8x, inpatient 2.6x and outpatient 2.8x. The most common pneumonia treatment setting overall was in outpatient clinics (65% of all pneumonia events). Among individuals with DM, inpatient treatment was used more frequently than ED (24% *vs.* 18%), while individuals without DM were treated similarly between ED and inpatient (17% *vs.* 15%).

The annual total expenditure for pneumonia events in 2014 was $3 billion and $9 billion in the DM population and the non-DM population, respectively. This was mostly driven by inpatient costs, comprising 89% of all expenditures. The average cost per pneumonia event was higher among DM compared to non-DM in the inpatient setting ($11 931 *vs.* $7751; *P* < 0.001). Additionally, the average cost expenditures per pneumonia event were similar when managed in the outpatient ($344 *vs.* $87; *P* = 0.314) and ED ($1072 *vs.* $883; *P* = 0.449) for DM and non-DM individuals, respectively.

## Discussion

This large cross-sectional study describes the pneumonia prevalence, risk factors and healthcare utilisation patterns for individuals with DM in the USA. This study demonstrated that individuals with DM, especially those with DM-complications, have a higher likelihood for being treated with pneumonia and are associated with a disproportionate economic burden.

Individuals with DM are considered at higher risk for infections [4, 6, 8]. In a systematic review, individuals with DM were found to have a 40% higher risk for community-acquired pneumonia (CAP) and 2.3-fold higher risk for pneumococcal pneumonia [5]. In a study conducted by The Emerging Risk Factors Collaboration, it was found that individuals with DM had a 1.7x higher mortality risk from pneumonia compared to those without DM [9]. Yende *et al*. determined that patients with DM had a higher risk of mortality post-CAP [10]. Finally, Martins *et al*. demonstrated that DM patients had a higher prevalence, longer hospital stay and higher risk of mortality from CAP than non-DM patients [11]. Our study findings further support the notion that pre-existing DM is associated with a higher risk of acquiring pneumonia. This study also identified that the population rate for pneumonia among those with DM complications was doubled compared to those without complications, implying that DM complications may be an additional risk factor for pneumonia in this population.

Furthermore, this study found that individuals with DM had a higher rate of seeking care for pneumonia compared to individuals without DM across all three treatment settings. The primary setting for the management of pneumonia was in outpatient clinics, comprising of ~70% of all visits for pneumonia. However, individuals with DM were more likely to be hospitalised for pneumonia compared to non-DM. When examining individuals with DM, those with DM-complications had higher rates of seeking care for pneumonia across all treatment settings. Furthermore, the estimated total annual expenditure for pneumonia was $12 billion in 2014 with ~90% driven by inpatient costs. A 2007 retrospective database analysis found that the presence of DM was associated with a 70% higher average healthcare cost for pneumonia. Inpatient DM patients incurred an additional $8404 compared to its non-DM counterparts [12]. A 2008–2014 commercial claim study determined that the overall mean pneumonia hospitalisation cost was $10 963 [13]. Comparatively, this study describes a disproportionately higher average cost per pneumonia hospitalisation among DM compared to non-DM.

This study identified female sex as a risk factor for pneumonia among individuals with DM. Past studies evaluating sex as a risk factor for pneumonia are limited and inconsistent. Recent literature suggests that male sex increases the incidence and severity of pneumonia. Male patients have demonstrated to have a higher risk for lower respiratory tract infections (LRTIs) and higher hospital mortality from pneumonia [14, 15]. The pneumonia severity indices include male sex as a risk factor in assessing the mortality risk and disposition [16]. However, these studies do not account for other factors including underlying effects of DM and sex [17]. Manicardi *et al*. found that women with DM had consistently higher haemoglobin A1c than males despite the same treatment intensity and goals, suggesting a pathophysiological difference between sex [18]. Further studies are needed to understand potential sex differences in DM and risk for pneumonia [19].

Factors associated with DM-complications were found to be associated with pneumonia among individuals with DM. Kornum *et al*. identified that having DM for greater than 10 years was associated with a 1.4x risk of having a pneumonia-related hospitalisation [20]. Studies evaluating infection risk among those with DM-complications, primarily those with diabetic nephropathy, have shown that the presence of DM-complications is associated with higher pneumonia incidence rates and poorer prognosis [21, 22]. Our study further supports that individuals with DM-complications are more likely to be hospitalised. Together, these findings suggest that targeted initiatives for this population are needed due to their significantly higher risk for pneumonia. Current pneumonia risk assessment scores do not factor in DM, nor associated complications, into determining risk and treatment [23].

A large number of studies have demonstrated that preventative pneumococcal and influenza vaccination improved clinical and economic outcomes, particularly among DM individuals and older adults [24–28]. However, vaccination rates continue to remain suboptimal. We found that only 49% of adults reported receiving an influenza vaccine during the past year and 61% reported receiving a pneumococcal vaccine. These rates are consistent with those described in other reports ranging from 20% to 38% for high-risk groups under the age of 65 years and 60–72% for those over the age of 65 years [29, 30]. These low vaccination rates are likely due to multiple factors including misconceptions and lack of education about pneumococcal vaccinations among providers and the public [31]. A recent survey administered by the American Diabetes Association showed that only 35% of DM individuals believed that they were at high risk for pneumonia. This underscores a greater need for proper patient education [32].

There were limitations to this study. First, we used CCS codes to identify pneumonia occurrences. Relying on coding for pneumonia rates is inherently subject to bias introduced by potential misclassification and coding errors. While LRTIs are frequently managed by nurse practitioners, physician assistants and other non-physician clinicians, this sample did not include non-physician clinician office-based visits. This might have underestimated the true burden of pneumonia in the ambulatory or outpatient setting. Moreover, this was a cross-sectional study design and might be susceptible to ecological fallacy bias. The prevalence data were based on total events and does not provide patient-specific information. Therefore, it is impossible to determine disease severity, microbiological aetiolgy, or other radiology or laboratory values to validate the diagnosis of pneumonia. Other potential confounders not available in this dataset were not considered in the analysis. However, to the best of our knowledge, this is the first large study to provide a holistic perspective of the burden of pneumonia and the care-seeking patterns in individuals with and without DM in the USA. This study also provides insights into previously unconsidered factors for individuals with DM who experience pneumonia.

In conclusion, individuals with DM have a higher risk for increased healthcare utilisation patterns compared to non-DM individuals for the management of pneumonia. Further studies are needed to examine specific risk factors for this population and their impact on pneumonia treatment decision-making and infection prevention efforts.
